# Individualized Management of Patients with Coronary Artery Disease

**DOI:** 10.3390/jpm12081243

**Published:** 2022-07-29

**Authors:** Jacek Bil

**Affiliations:** Department of Invasive Cardiology, Centre of Postgraduate Medical Education, 02-507 Warsaw, Poland; biljacek@gmail.com; Tel.: +48-47-722-11-00

We can characterize personalized medicine as a compilation of diagnostic and screening modalities to better manage individual patients, their diseases, or their predisposition toward various disorders. Remarkable progression in genomic studies has been achieved in recent decades, from the characterization of the deoxyribonucleic acid (DNA) double helix by Watson and Crick in 1953 to the completion of the Human Genome Project in 2003. In addition, the Human Genome Project and the International HapMap Project have provided a tremendous amount of information received from DNA analysis.

Moreover, individualized medicine represents another stage in the development of personalized medicine. While personalized medicine targets a specific group of patients, individualized medicine tackles the particular circumstances of a single person. Thus, individualized medicine goes one step further and can be perceived as progress in the specificity of personalized medicine. Individualized medicine is not only focused on genes but also looks at the full range of a person’s unique nature, including biological, physiological, and anatomical characteristics.

At many points in one’s lifetime, an individual’s unique characteristics play an increasingly important role. Before conception, couples can screen themselves to identify any essential recessive alleles. Then, an expectant mother can find out if the fetus has any chromosomal abnormalities, and the sex can be determined. Using the heel stick method, newborns can be screened with genome sequencing for many severe conditions (e.g., cystic fibrosis). Later, omics data can provide information on an individual’s susceptibility to various diseases for which prevention strategies can be proposed, such as diabetes or some types of cancer. Finally, in case of sudden death, molecular sequencing during autopsy (together with family survivors) can provide the cause of death and possibly prevent untimely or avoidable deaths in future generations [1].

In patients with coronary artery disease, conventional risk factors cannot explain even half of the cases of morbidity and mortality. Additionally, conventional and novel risk factors have a partially genetic origin. Various studies reported that treatment algorithms supported by consensus guidelines might lead to a scenario where a significant percentage of patients (e.g., patients with no Apo E genotype) do not receive optimal care. Therefore, an approach defined as “one diet and standard drug regimen fits all” is often not the best option.

All of these factors indicate that the transformation of medicine in the 21st century can be named “P4 Medicine”, i.e., Predictive, Personalized, Preemptive, and Participatory. Moreover, genome records could enable physicians to make treatment decisions based on patients’ genotypes. This would allow individuals to make appropriate lifestyle choices and enable them to use appropriate drugs.

Personalized medicine is developing quickly, and massive amounts of new data are emerging. This might enable proper risk assessments, diagnoses, prevention, and therapy specifically tailored to an individual’s unique characteristics, thus enhancing their quality of life and public health. In the previous Special Issue regarding personalized medicine in coronary artery disease, we published eight valuable papers showing how an approach tailored to individual patients can improve outcomes. They included one review paper and seven research studies.

In a review paper, Rychter et al. summarized the knowledge on carotid intima media thickness (IMT) measurement among obese patients as well as options for an individualized approach to decrease IMT in this group, taking into consideration diet and behavioral changes [2].

Original studies focused on options to identify markers allowing more individual risk stratification as well as the optimization of pharmacotherapy and secondary prevention. Lizama et al. verified the prevalence of kidney disease, potassium levels, and cardiovascular risk factors in patients with coronary artery disease [3]. They showed that coronary atherosclerotic lesions with ≥ 30% diameter stenoses were more frequent in males, smokers, and in subjects with arterial hypertension and/or diabetes. Interestingly, aside from glucose and creatinine levels, the potassium concentration was also linked with the presence of coronary lesions ≥ 30% in males.

Meanwhile, Gurzau et al. studied whether interleukin 6 (IL-6) and endothelin 1 (ET-1) can play a role in microvascular angina in women [4]. The authors reported an increased IL-6 level comparable in patients with microvascular angina, one-vessel, and two-vessel coronary artery disease, but this level was significantly lower than that in women with three-vessel coronary artery disease. In microvascular angina patients, IL-6 correlated with the NYHA IV functional class. Surprisingly, ET-1 correlated with left ventricular systolic dysfunction. The authors suggested that ET-1 can be perceived as a diagnostic marker in women with microvascular angina who cannot be tested invasively.

Karagiannidis et al. published a very important paper regarding serum ceramides in patients with STEMI [5]. The authors analyzed correlations between serum ceramide concentrations and micro-CT-quantified aspirated thrombus volume and angiographic outcomes. They disclosed that higher ceramide C16:0 levels substantially correlated with larger aspirated thrombus volume, larger intracoronary thrombus burden, and worse pre- and post-procedural TIMI flow. Moreover, ceramides C24:0 and C24:1 were also significantly associated with a larger intracoronary thrombus burden. Therefore, the quantification of serum ceramides might improve the risk-stratification of patients with STEMI and facilitate an individualized approach in clinical practice. Additionally, Kern et al. reported on blood biomarkers. They assessed the impact of serum sclerostin levels in an almost decade-long ongoing follow-up in subjects undergoing coronary angiography but with no chronic kidney disease ≥ 3 stage [6]. In the high-sclerostin group (≥median), the authors reported statistically significant higher MACE and death rates: 25.2% vs. 43.1% (HR 1.75, 95% CI 1.1–2.10, *p* = 0.02) and 14.6% vs. 26.5% (HR 1.86, 95% CI 1.02–3.41, *p* = 0.049), respectively. Similar findings were disclosed in patients with chronic coronary syndrome and SYNTAX 0–22. These results suggest that sclerostin assessment might be useful in risk stratification, and subjects with higher sclerostin levels might have a worse prognosis.

Additionally, an interesting network meta-analysis was prepared by Grajek et al. They showed in a meta-analysis including 81.943 patients that 110 mg of dabigatran b.i.d. increased the myocardial infarction (MI) risk more than warfarin, apixaban, edoxaban, or rivaroxaban [7]. Additionally, 150 mg of dabigatran b.i.d. increased the MI risk more than warfarin, apixaban, and rivaroxaban. The authors also determined that rivaroxaban had a 90% probability of being perceived as the best therapy for MI prevention, whereas 110 mg of dabigatran had an 8.2% probability. However, 150 mg of dabigatran b.i.d was the most effective option in stroke prevention (94% probability). The authors stated that each novel oral anticoagulant (NOAC) is linked with a different MI risk. Moreover, they suggested that FXa inhibitors should be chosen as the first-line NOACs in patients with atrial fibrillation and coronary artery disease.

Szmigielska et al. focused on the differences in cardiac rehabilitation outcomes between women and men [8]. They showed that the most significant benefit was observed in men, women under 55 years, women with LVEF 41–49%, and women with single-vessel coronary artery disease. Outpatient cardiac rehabilitation was less beneficial in women, especially those over 55, with two- or three-vessel disease, or with LVEF < 50%. In women with coronary artery disease, 8 weeks of 45 min interval training, with sessions taking place three times a week, was not sufficient to enhance the exercise capacity to the extent that was considered a predictor of mortality risk reduction. Finally, Schnaubelt et al. determined whether ultra-sensitive phonocardiography, already used to rule out stable coronary artery disease, was feasible to use in the Emergency Department setting [9].

Herein, we introduced the second installment of our Special Issue entitled “Personalized Medicine for Coronary Artery Disease: Diagnosis, Prevention, and Treatment” [10]. Therefore, we invite researchers to submit their original studies, reviews, or meta-analyses on the individualization of the prevention, diagnosis, and treatment of coronary artery disease as well as coronary artery disease complications such as myocardial infarction or heart failure. We are soliciting not only genetic studies but all papers regarding broadly understood personalized or individualized medicine, including manuscripts devoted to novel drugs, medical devices, or tailored approaches dedicated to subsets of our patients.

## Data Availability

Not applicable.

## References

[1] Topol E.J. (2014). Individualized medicine from prewomb to tomb. Cell.

[2] Rychter A.M., Naskręt D., Zawada A., Ratajczak A.E., Dobrowolska A., Krela-Kaźmierczak I. (2021). What Can We Change in Diet and Behaviour in Order to Decrease Carotid Intima-Media Thickness in Patients with Obesity?. J. Pers. Med..

[3] Lizama P.M., Ríos D.L., Cachinero I.S., Lopez-Egea A.T., Camps A., Belzares O., Pacheco C., Cerro C., Wehinger S., Fuentes E. (2021). Association of Kidney Disease, Potassium, and Cardiovascular Risk Factor Prevalence with Coronary Arteriosclerotic Burden, by Sex. J. Pers. Med..

[4] Gurzău D., Sitar-Tăut A., Caloian B., Guşetu G., Comşa H., Frîngu F., Zdrenghea D., Pop D. (2021). The Role of IL-6 and ET-1 in the Diagnosis of Coronary MicroVascular Disease in Women. J. Pers. Med..

[5] Karagiannidis E., Papazoglou A.S., Stalikas N., Deda O., Panteris E., Begou O., Sofidis G., Moysidis D.V., Kartas A., Chatzinikolaou E. (2021). Serum Ceramides as Prognostic Biomarkers of Large Thrombus Burden in Patients with STEMI: A Micro-Computed Tomography Study. J. Pers. Med..

[6] Kern A., Stompór T., Kiewisz J., Kraziński B.E., Kiezun J., Kiezun M., Górny J., Sienkiewicz E., Gromadziński L., Onichimowski D. (2021). The Impact of Sclerostin Levels on Long-Term Prognosis in Patients Undergoing Coronary Angiography: A Personalized Approach with 9-Year Follow-Up. J. Pers. Med..

[7] Grajek S., Kałużna-Oleksy M., Siller-Matula J.M., Grajek M., Michalak M. (2021). Non-Vitamin K Antagonist Oral Anticoagulants and Risk of Myocardial Infarction in Patients with Atrial Fibrillation with or without Percutaneous Coronary Interventions: A Meta-Analysis. J. Pers. Med..

[8] Szmigielska K., Jegier A. (2022). Clinical Outcomes of Cardiac Rehabilitation in Women with Coronary Artery Disease—Differences in Comparison with Men. J. Pers. Med..

[9] Schnaubelt S., Eibensteiner F., Oppenauer J., Kornfehl A., Brock R., Poschenreithner L., Du N., Baldi E., Schlager O., Niessner A. (2022). The Feasibility of Ultra-Sensitive Phonocardiography in Acute Chest Pain Patients of a Tertiary Care Emergency Department (ScorED Feasibility Study). J. Pers. Med..

[10] https://www.mdpi.com/journal/jpm/special_issues/Personalized_Coronary_Treatment.

